# High fat diet exacerbates murine psoriatic dermatitis by increasing the number of IL-17-producing γδ T cells

**DOI:** 10.1038/s41598-017-14292-1

**Published:** 2017-10-26

**Authors:** Satoshi Nakamizo, Tetsuya Honda, Akimasa Adachi, Takahiro Nagatake, Jun Kunisawa, Akihiko Kitoh, Atsushi Otsuka, Teruki Dainichi, Takashi Nomura, Florent Ginhoux, Koichi Ikuta, Gyohei Egawa, Kenji Kabashima

**Affiliations:** 1 Department of Dermatology Kyoto University Graduate School of Medicine, Kyoto, 606-8507 Japan; 2 Singapore Immunology Network (SIgN) and Institute of Medical Biology (IMB), Agency for Science, Technology and Research (A*STAR), 8A Biomedical Grove, IMMUNOS Building, Biopolis, 138648 Singapore; 3 Laboratory of Vaccine Materials, National Institutes of Biomedical Innovation, Health and Nutrition, Osaka, 567-0085 Japan; 4 Laboratory of Biological Protection, Department of Biological Responses, Institute for Virus Research, Kyoto University, Kyoto, 606-8507 Japan; 5 PRESTO, Japan Science and Technology Agency, 4-1-8 Honcho, Kawaguchi, Saitama, 332-0012 Japan

## Abstract

Psoriasis is a common, chronic inflammatory skin disease characterized by epidermal hyperplasia via the IL-23/IL-17 axis. Various studies have indicated the association between obesity and psoriasis, however, the underlying mechanisms remains unclarified. To this end, we focused on high-fat diet (HFD) in this study, because HFD is suggested as a contributor to obesity, and HFD-fed mice exhibit exacerbated psoriatic dermatitis. Using murine imiquimod (IMQ)-induced psoriasis and HFD-induced obesity models, we have revealed a novel mechanism of HFD-induced exacerbation of psoriatic dermatitis. HFD-fed mice exhibited aggravated psoriatic dermatitis, which was accompanied with increased accumulation of IL-17A-producing Vγ4^+^ γδ T cells in the skin. HFD also induced the increase of Vγ4^+^ γδ T cells in other organs such as skin draining lymph nodes, which preceded the increase of them in the skin. In addition, HFD-fed mice displayed increased expression of several γδ T cell-recruiting chemokines in the skin. On the other hand, *ob/ob* mice, another model of murine obesity on normal diet, did not exhibit aggravated psoriatic dermatitis nor accumulation of γδ T cells in the dermis. These results indicate that HFD is a key element in exacerbation of IMQ-induced psoriatic dermatitis, and further raise the possibility of HFD as a factor that links obesity and psoriasis.

## Introduction

The prevalence of obesity in developed countries has approximately doubled over the last two decades^1^. Although the actual reasons for such an increase remain unknown, increased intake of high-fat diet (HFD), containing abundant saturated fatty acids or trans-unsaturated fatty acids, is suggested as one of the contributors^2^. Recently, it has widely been accepted that obesity is associated with the development of various inflammatory diseases including psoriasis, a common, chronic, interleukin (IL)-17-mediated inflammatory skin disease^3–5^.

Various studies have shown the association between obesity and the prevalence or severity of psoriasis^6–8^. Although the causal relationship between obesity and psoriasis remains unclarified, a number of clinical studies suggest that obesity is an upstream event that causes or exacerbates psoriasis. For example, it has been reported that childhood-onset obesity predisposes an individual to both psoriasis and psoriatic arthritis^9^, and that a rapid remission of psoriasis is induced after bariatric surgery^6^. In addition, progressive weight loss can induce significant improvements in the severity of psoriasis^10^. These reports suggest that obesity is a trigger or causal factor of psoriasis.

Several possibilities are proposed as pathological mechanisms that link obesity and psoriasis^11–14^. Among them, HFD is suspected as one of such factors, since increased intake of HFD (containing saturated fatty acids or trans-unsaturated fatty acids) is suggested as a contributor to obesity^2,15^. In addition, several studies using a mouse psoriasis model indicated the exacerbation of psoriatic dermatitis in mice fed with HFD^12,13^. It has been reported that HFD induces IL-1β and IL-18 production from macrophages by activating the NLRP3 (nucleotide-binding domain, leucine-rich repeats-containing family, pyrin domain-containing-3) inflammasome pathway^16^, which are suggested to facilitate inflammatory cell infiltration into the skin, and exacerbates psoriatic dermatitis. Thus, HFD is suggested to connect the association between obesity and psoriasis. Therefore, elucidation of molecular mechanisms that link HFD and psoriasis is an important and attractive research/clinical topic that needs further investigation.

To clarify a novel mechanism by which HFD affects the development of psoriasis, we used a mouse HFD-induced obese model and a mouse psoriasis model. We used a saturated fatty acids-rich HFD for the experiment. We determined that the intake of HFD causes an increased accumulation of IL-17A-producing γδ T cells in the skin, which leads to exacerbation of psoriatic dermatitis. HFD also induced a systemic increase of γδ T cells, the infiltration of which appeared to be facilitated by γδ T cell-recruiting chemokines in the skin induced by HFD. *ob/ob* mice, another obesity model on normal diet (ND), did not exhibit aggravated psoriatic dermatitis. Our results suggest a novel pathway for the HFD-induced exacerbation of psoriasis, and may propose a therapeutic modality for psoriasis.

## Results

### HFD is associated with aggravated psoriatic dermatitis upon imiquimod (IMQ) treatment

**Figure 1:**
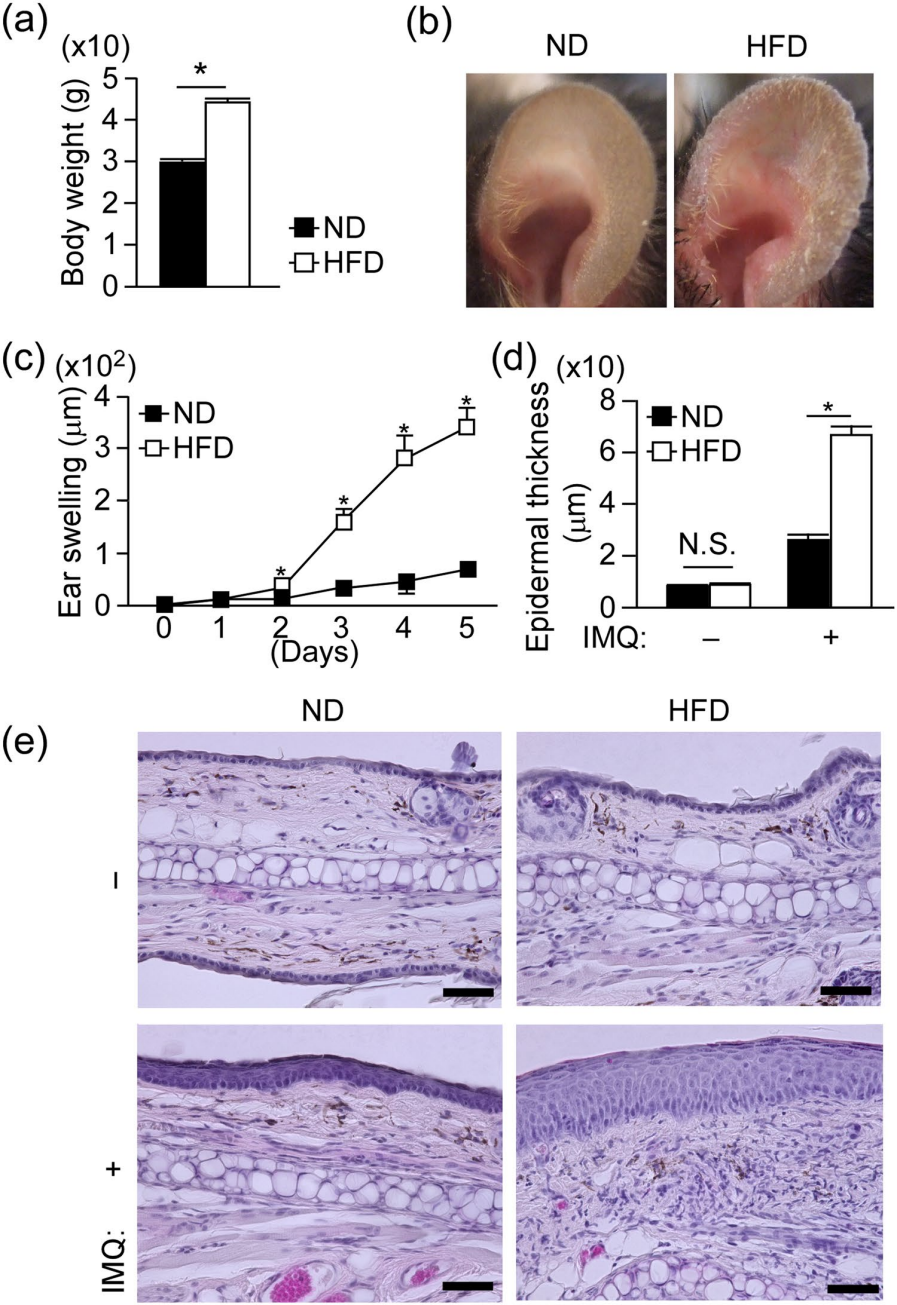
High-fat diet-induced obesity exacerbates imiquimod-induced psoriatic dermatitis. (**a**) Comparison of body weight between normal diet (ND)- and high fat diet (HFD)-fed mice 10 weeks after feeding. (**b**–**d**) Imiquimod (IMQ)-induced psoriatic dermatitis in wild-type (WT) mice fed with either a ND- or HFD-diet. Mice were treated with IMQ for five consecutive days. (**b**) Representative pictures of psoriatic dermatitis in ear skin. (**c**) Time course of ear swelling response. (**d**) Epidermal thickness change with or without IMQ treatment for five days. (**e**) Representative pictures of H&E-stained sections of the ear skin in ND- and HFD-fed mice with or without IMQ treatment for five days. Samples were collected at 24 h after the last IMQ treatment. Scale bars = 50 μm. Results are expressed as the mean ± SD (**c**) and SEM (**a**,**d**). *p*-values were obtained by Mann-Whitney-U-test. *p ≤ 0.05. Data are from one experiment, representative of six independent experiments with three to four mice (**a**), three experiments with three to four mice (**b**–**e**).

To examine a novel mechanism by which HFD promotes the development of psoriasis, we first confirmed the HFD-induced exacerbation of psoriatic dermatitis using a HFD-fed obese mice and an imiquimod (IMQ)-induced psoriasis model^17^. We fed C57BL/6 mice with a HFD, in which fats occupy 60% of the total calories, for 10 weeks and induced obesity (Fig. 1a). Control mice were fed with a normal diet (ND) in which fats occupy 13% of the total calories. Then, we applied IMQ onto the ear skin for five days, and examined the inflammatory responses. Mice fed with HFD exhibited significantly increased ear swelling compared to ND-fed mice (Fig. 1b,c) as reported previously^12^. Histological analysis revealed enhanced epidermal hyperplasia and mixed mononuclear infiltrates in the skin of IMQ-treated HFD-fed mice, compared with those of ND-fed mice (Fig. 1d,e). No obvious difference was detected in epidermal thickness between ND- or HFD-fed mice without IMQ treatment (Fig. 1d). Thus, we established an appropriate model to analyze the underlying mechanisms on HFD-induced exacerbation of psoriasis.

### HFD induces accumulation of IL-17A producing Vγ4^+^ γδ T cell in the skin

Various immune cells, especially neutrophils, dendritic cells (DCs), and γδ TCR^mid^ γδ T cells, cooperatively contribute to the development of IMQ-induced psoriatic dermatitis^18^. Therefore, we next examined the influence of HFD on skin immune cell composition. Ear skin was digested as described in Material and Methods, and single cell suspensions were subjected to flow cytometric analysis (Supplementary Figure 1). It should be taken into account that the number of cells that locate in epidermis might be underrepresented with this digestion protocol, since this protocol was optimized for the digestion of dermis, but not of epidermis.

**Figure 2:**
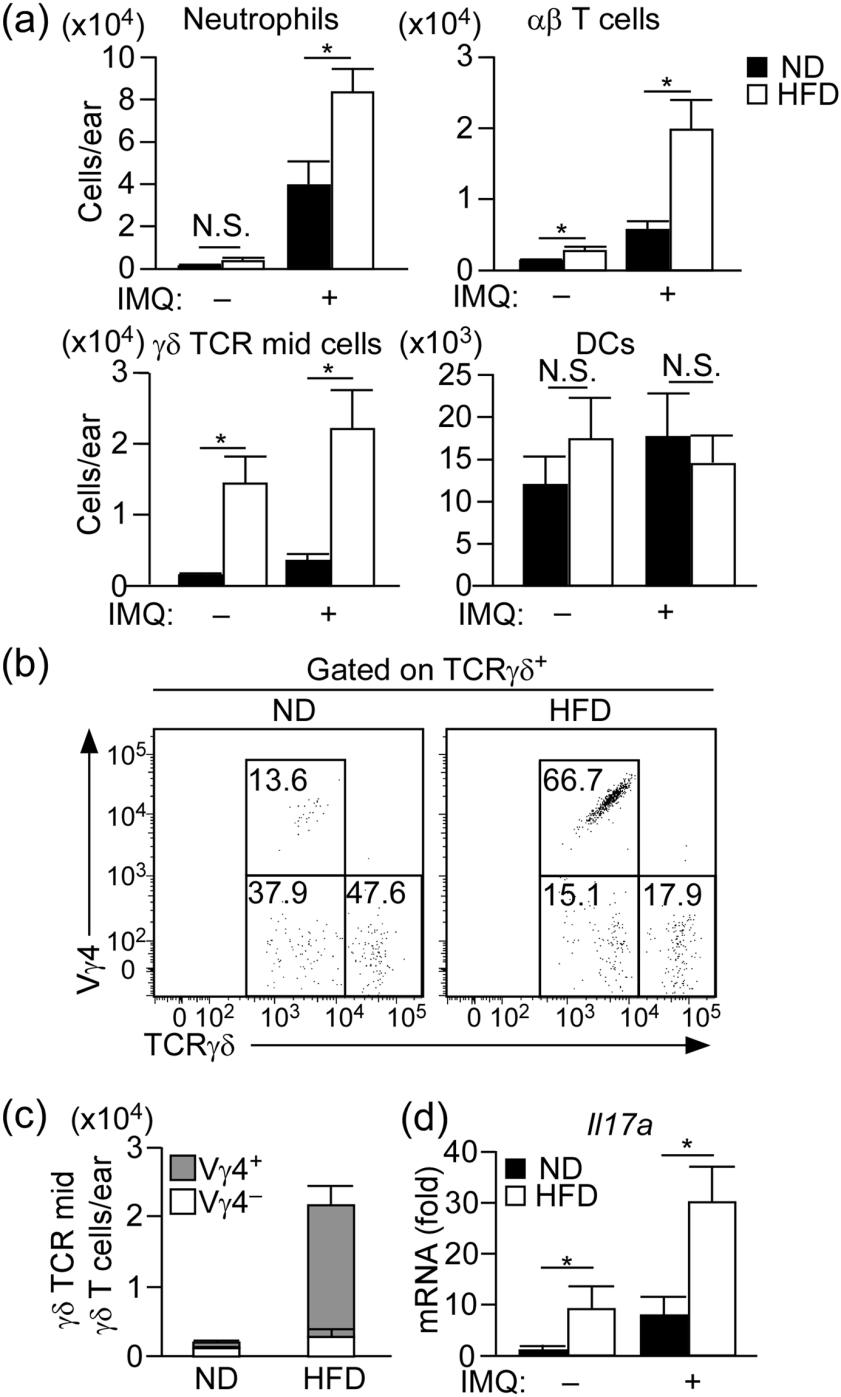
Vγ4^+^ γδ T cells are accumulated in the skin of HFD-fed mice. (**a**) Flow cytometric analysis of the number of immune cells in the whole ear skin of ND- and HFD-fed mice with or without IMQ treatment for five days. Samples were collected at 24 h after the last IMQ treatment. Neutrophils: CD45^+^ Ly6G^+^, αβ T cells: CD45^+^ TCRβ^+^, γδ TCR^mid^ γδ T cells: CD45^+^ TCRγδ ^mid^, dendritic cells (DCs): CD45^+^ CD11c^+^ MHC class 2^high^. (**b**,**c**) Flow cytometric analysis of the γδ TCR^mid^ γδ T cells subset in the steady state whole ear skin. (**d**) Fold induction of *Il17a* mRNA in the whole ear skin of ND- or HFD-fed mice with or without IMQ treatment for five days, as analyzed by quantitative RT-PCR (qRT-PCR). Samples were collected at 24 h after the last IMQ treatment. Results are presented relative to those of ND. The average mRNA expression level in ND-fed mice is set as 1. Results are expressed as the mean ± SEM. *p*-values were obtained by Mann-Whitney-U-test. *p ≤ 0.05. Data are pooled from three experiments with three to four mice (**a**). Data are from one experiment, representative of six independent experiments with three to four mice (**b**,**c**) Data are pooled from three independent experiments with three to five mice each group (**d**).

The numbers of DCs were comparable between IMQ-treated ND- and HFD-fed mice. However, the numbers of neutrophils, αβ T cells and γδ TCR^mid^ γδ T cells (which consist of Vγ4^+^ γδ T cell subset and Vγ4^−^ γδ T cell subset) were significantly increased in the ear skin of IMQ-treated HFD-fed mice (Fig. 2a). It is of note that αβ T cells and γδ TCR^mid^ γδ T cells were increased in the HFD-fed mice even in the steady state (Fig. 2a). Particularly, the number of γδ T cells was 10 times higher than that of ND-fed mice. Most of the increased-γδ T cell subset was Vγ4^+^ γδ T cells (Fig. 2b,c), which is one of the main IL-17A-producing cell subset in the IMQ-induced psoriasis model^19^. Consistently, the mRNA expression of IL-17A in the steady and inflammatory state skin of HFD-fed mice was significantly increased than that in ND-fed mice (Fig. 2d).

**Figure 3:**
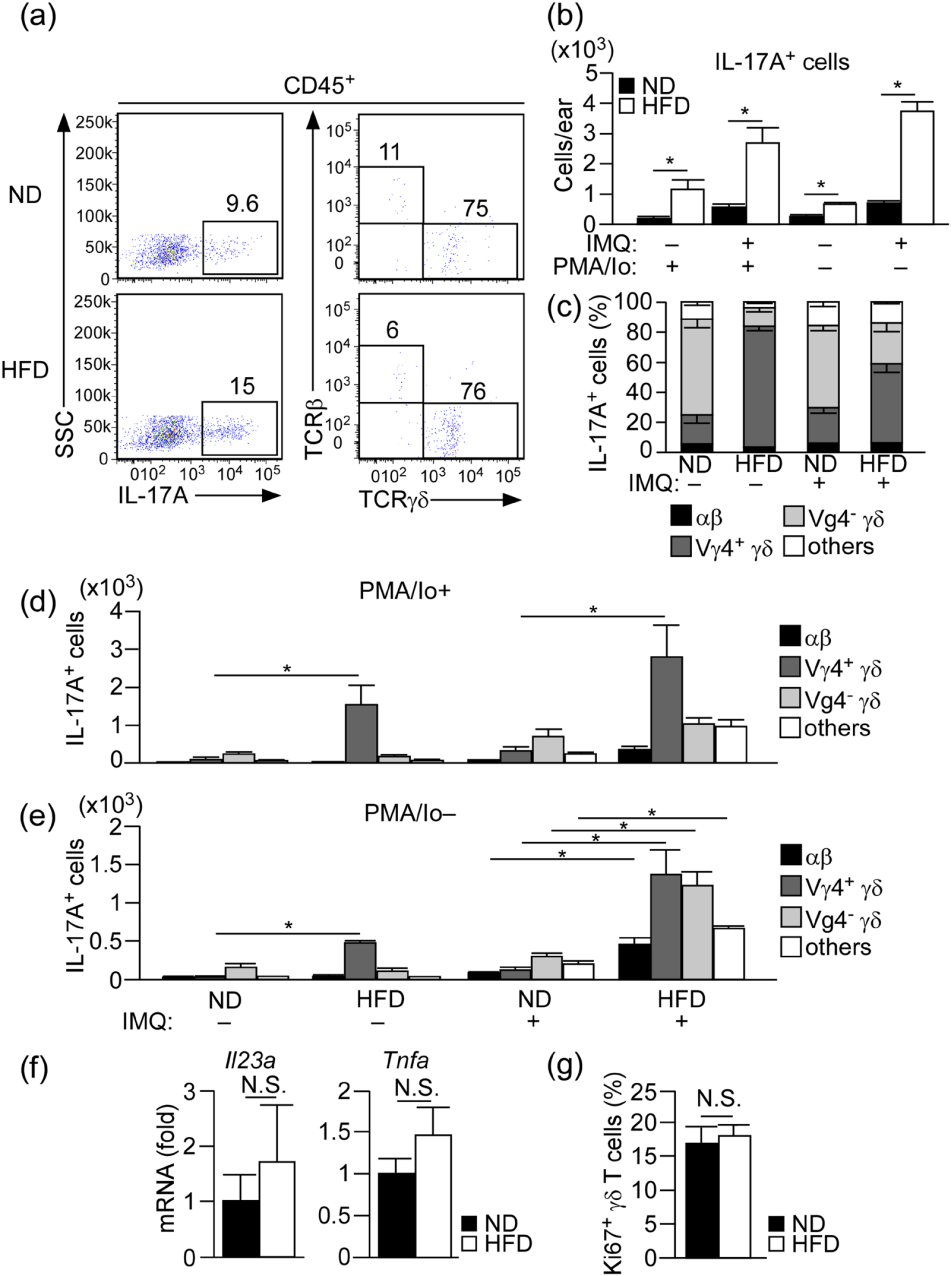
Increase of IL-17A-producing cells in the skin of HFD-fed mice in both steady and inflammatory states. (**a**) Flow cytometric analysis of IL-17A-producing cells in the whole ear skin of HFD- or ND-fed mice in the steady state. The single cell suspensions were stimulated with phorbol myristate acetate (PMA) and ionomycin in the presence of brefeldin A for 3 h before intracellular staining. Left panels indicate cells gated on CD45^+^, and right panels indicate cells gated on IL-17A^+^ in the left panels. (**b**–**e**) Statistical analysis of the number of total IL-17A-producing cells (**b**) and the percentage (**c**) and number/composition (**d**,**e**) of IL-17A-producing cells. Samples were collected at 24 h after the last IMQ treatment, and the ear skin was digested with collagenase in the presence or absence of brefeldin A. Single cell suspensions were either stimulated with PMA and ionomycin in the presence of brefeldin A for 3 h before intracellular staining (**b**–**d**) or directly subjected to intracellular staining (**b**,**e**). (**f**) Fold induction of *Il23a* and *Tnfa* mRNA in the whole ear skin of ND- or HFD-fed mice in the steady state, as analyzed by quantitative RT-PCR. Results are presented relative to those of ND. The average mRNA expression level in ND-fed mice is set as 1. (**g**) Flow cytometric analysis of the ratio of Ki67^+^ TCRγδ^+^ cells in the whole ear skin in the steady state. Results are expressed as the mean ± SEM. *p*-values were obtained by Mann-Whitney-U-test (**b**,**f**,**g**) and one-way ANOVA (**d**,**e**). *p ≤ 0.05. Data are from one experiment, representative of four independent experiments with three to four mice (**a**), two experiments with four mice (**g**). Data are pooled from two experiments with three to four mice (**b**–**e**), three experiments with three mice (**f**).

Therefore, we next examined the number and the composition of IL-17A-producing cells in the skin of ND- and HFD-fed mice with or without IMQ treatment. Using a flow cytometry, we first examined the number of cells that have a potential to produce IL-17A, by stimulating the skin cells with phorbol myristate acetate (PMA) and ionomycin *in vitro*. The number of total IL-17A-producing cells was significantly higher in HFD-fed mice compared with that in ND-fed mice in both the steady and inflammatory states **(**Fig. 3a,b **)**. The majority of IL-17A-producing cells were γδ TCR^mid^ γδ T cells **(**Fig. 3c **)**, and approximately 15% of them produced IL-17A, in both ND- and HFD- fed mice in steady and inflammatory state (Supplementary Figure 2a,b (left panel)). In the IL-17A-producing γδ TCR^mid^ γδ T cells, most of them were Vγ4^+^ γδ T cells in the steady state of HFD-fed mice, while Vγ4^−^ γδ T cells were the major IL-17A-producing γδ TCR^mid^ γδ T cells in ND-fed mice **(**Fig. 3c,d). In the IMQ-treated skin of HFD-fed mice, approximately 50% of IL-17A-producing cells were Vγ4^+^ γδ T cells, while the major cell population in ND-fed mice was a Vγ4^−^ γδ T cell subset **(**Fig. 3c,d).

We also examined the number of cells that produce IL-17A *in situ*. We digested the skin with collagenase in the presence of brefeldin A, and analyzed the number of IL-17A-producing cells by a flow cytometery. In the steady state skin, the majority of IL-17A-producing cells in HFD-fed mice were Vγ4^+^ γδ T cells, and the number was significantly higher than that in ND-fed mice (Fig. 3e). The major IL-17A-producing population in ND-fed mice was Vγ4^−^ γδ T cells. In IMQ-treated skin, the numbers of IL-17A-producing cells in HFD-fed mice were significantly increased in all the IL-17A-producing cell subsets, including Vγ4^+^ γδ T cells, Vγ4^−^ γδ T cells, αβ T cells, and non-γδ and αβ T cell subset, than those in ND-fed mice (Fig. 3e). Vγ4^+^ γδ T cells and Vγ4^−^ γδ T cells occupied most of the IL-17A-producing cells in HFD-fed mice (Fig. 3e). The percentage of IL-17A-producing γδ TCR^mid^ γδ T cells in total γδ TCR^mid^ γδ T cells was approximately 8% in every group **(**Supplementary Figure 2b (right panel)), however the mean fluorescence intensity (MFI) of IL-17A was significantly increased in inflammatory state compared with that in steady state in both ND- and HFD- fed mice **(**Supplementary Figure 2c).

IL-17A is a key cytokine for the development of psoriasis; however, an alternative pathway has been reported in the development of IMQ-induced psoriatic dermatitis in the absence of IL-17 receptor signaling^20^. To confirm the contribution of IL-17A in the exacerbated skin inflammation in HFD-fed mice, we applied IL-17A-deficient mice to the HFD-induced obese model and then to the IMQ-induced psoriasis model. IL-17A-deficient mice with HFD developed obesity to the similar extent to that of HFD-fed wild-type (WT) mice (Supplementary Figure 3a), and the increase of dermal γδ T cells was observed in both WT and IL-17A-deficient mice (Supplementary Figure 3b,c). However, HFD-induced exacerbation of ear swelling was completely abolished in IL-17A deficient mice (Supplementary Figure 3d). These results indicate that HFD-induced exacerbation of psoriatic dermatitis is dependent on IL-17A.

To examine the effect of HFD on γδ T cell proliferation in the skin, we checked the mRNA expression level of γδ T cell-proliferating factors such as *Il1a*, *Il6*, *Il23a*, *Tnfa* and *Tgfb1* in the skin, and found comparable expressions of these factors between HFD- and ND-fed mice **(**Fig. 3f and Supplementary Figure 4 **)**. Furthermore, flow cytometry analysis revealed a similar percentage of Ki67-positive-proliferating γδ T cells in the skin in both groups, suggesting that HFD does not directly induce the proliferation of γδ T cells in the skin **(**Fig. 3g **)**.

Taken together, these results suggest that HFD exacerbates IMQ-induced psoriatic dermatitis by increasing the number of IL-17A-producing γδ TCR^mid^ γδ T cells, especially Vγ4^+^ γδ T cells in the skin.

### HFD is required for the exacerbation of psoriatic dermatitis

**Figure 4:**
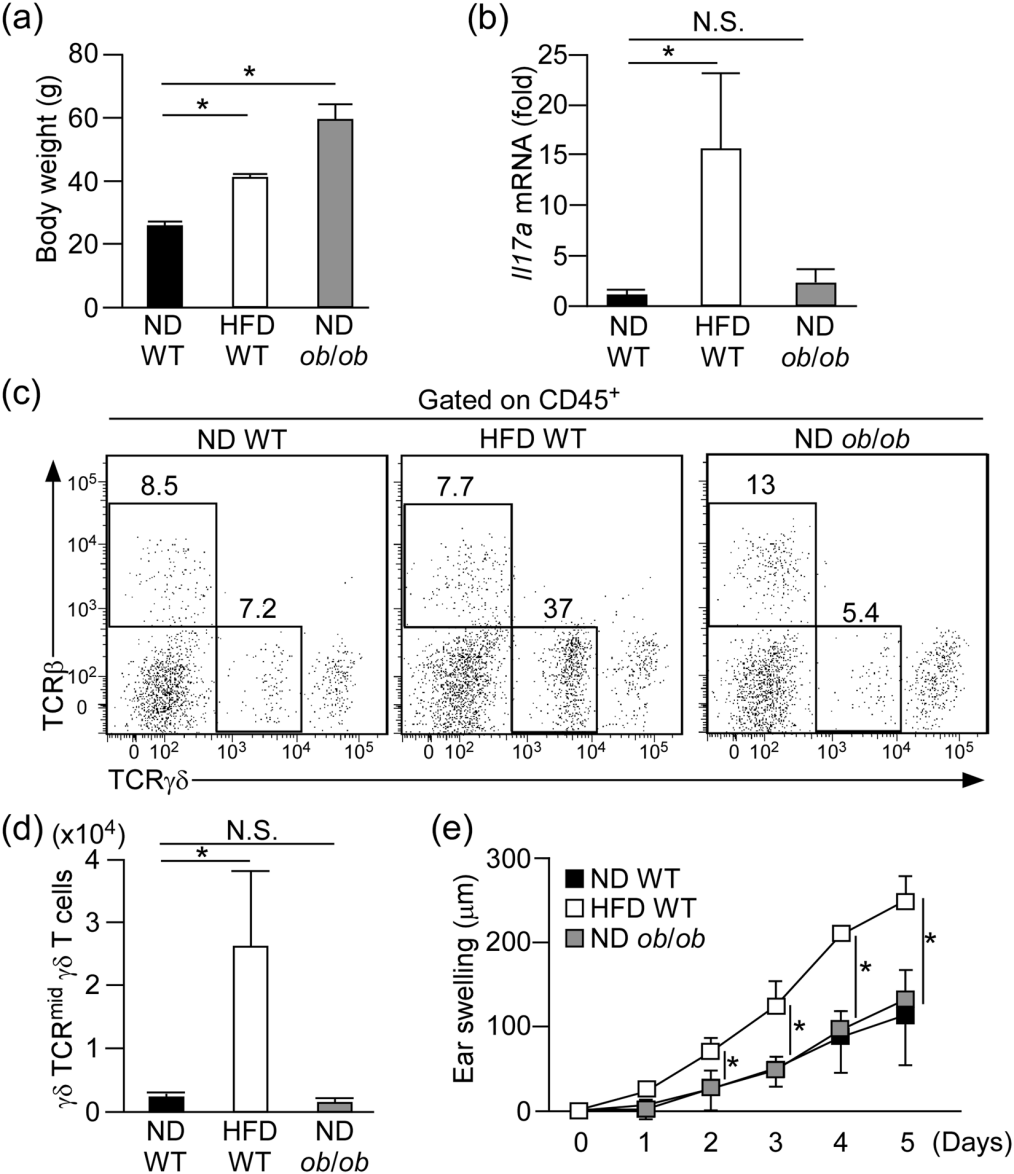
HFD is required for the exacerbation of psoriatic dermatitis. (**a**) Comparison of body weight in WT mice fed with either ND or HFD and *ob*/*ob* mice fed with ND. (**b**) Fold induction of *Il17a* mRNA in the skin of WT mice fed with ND or HFD and *ob*/*ob* mice fed with ND in the steady state, as analyzed by qRT-PCR. Results are presented relative to those of WT with ND. The average mRNA expression level in ND-fed WT mice is set as 1. (**c**,**d**) Flow cytometric analysis of T cell subsets (**c**) and the number of γδ TCR^mid^ γδ T cells (**d**) in the whole ear skin of WT mice fed with either ND or HFD and *ob*/*ob* mice fed with ND in the steady state. (**e**) IMQ-induced psoriatic dermatitis in WT mice fed with either ND or HFD and *ob*/*ob* mice fed with ND assessed as change in ear swelling. Results are expressed as the mean ± SD (**e**) and SEM (**a**–**c**). *p*-values were obtained by one-way ANOVA. *p ≤ 0.05. Data are from one experiment, representative of four independent experiments with three to four mice (**a**,**c**,**d**), three experiments with four mice (**e**). Data are pooled from three experiments with three to four mice (**b**).

To examine whether obesity without intake of HFD associates with exacerbated psoriatic dermatitis, we applied *ob*/*ob* mice, which develop obesity even on a ND as a result of increased appetite due to a deficiency of leptin^21^ (Fig. 4a), to the psoriasis model. In contrast to the results seen in HFD-fed mice, the *Il17a* expression and number of γδ TCR^mid^ γδ T cells in the skin were comparable between *ob*/*ob* mice and control lean mice (ND-fed WT mice) (Fig. 4b–d). In addition, the extent of the ear swelling response was comparable between *ob*/*ob* mice and control lean mice after IMQ treatment (Fig. 4e). These results suggest that HFD is required for the exacerbation of psoriatic dermatitis.

### HFD induces systemic increase of Vγ4^+^ γδ T cells

**Figure 5:**
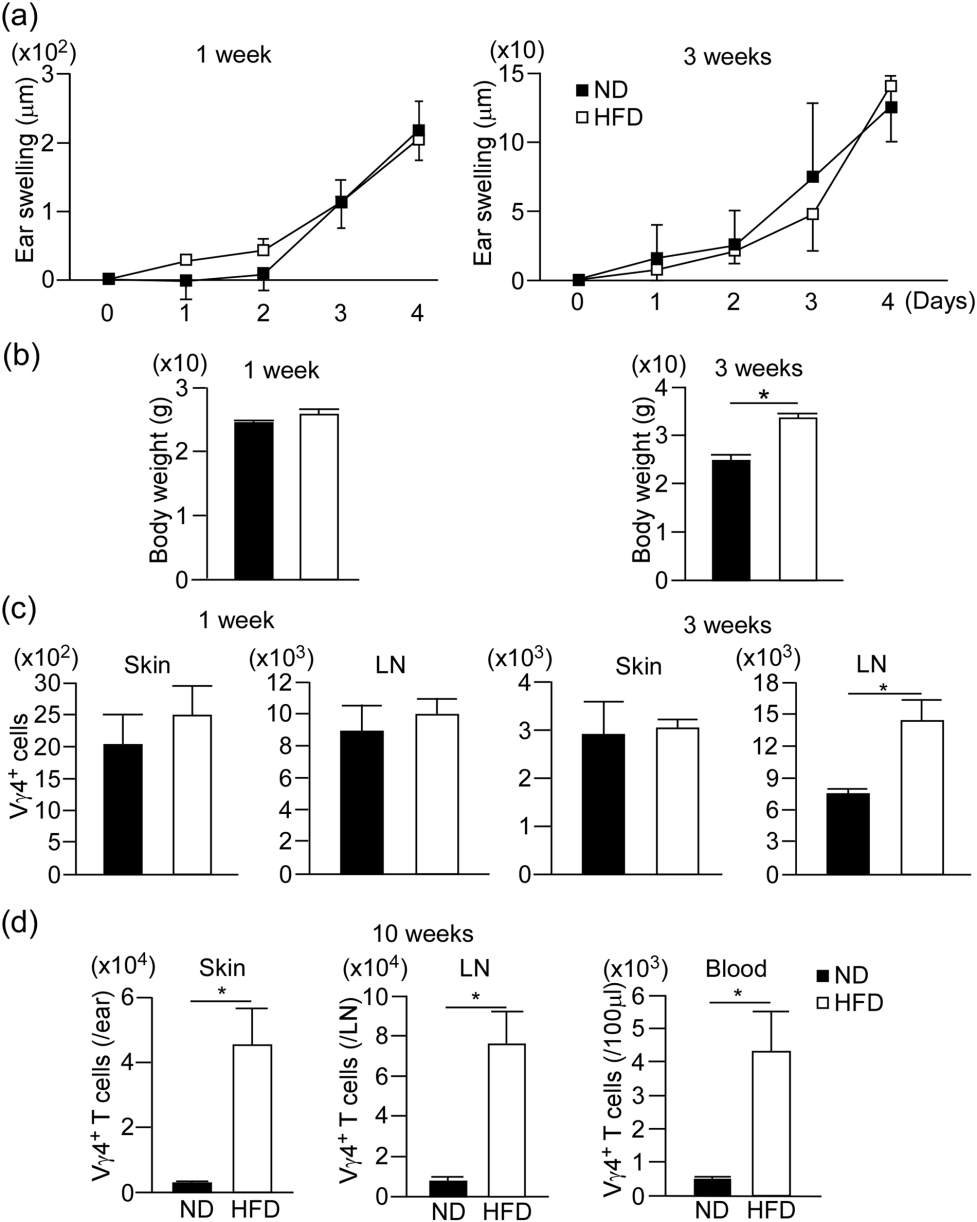
HFD induces the increase of Vγ4^+^ γδ T cells in the skin draining lymph node and blood. (**a**–**c**) Ear swelling response in IMQ-induced psoriasis model (**a**), body weight (**b**) and the number of Vγ4^+^ γδ T cells in the whole ear skin and the draining LNs (**c**) of steady state ND- and HFD-fed mice at 1 and 3 week after HFD-feeding. (**d**) Flow cytometric analysis of the number of Vγ4^+^ γδ T cells in the whole ear skin, cervical lymph node and peripheral blood of steady state ND- and HFD-fed mice at 10 week after HFD-feeding. Results are expressed as the mean ± SD (**a**), SEM (**b**–**d**). *p*-values were obtained by Mann-Whitney-U-test. *p ≤ 0.05. Data are from one experiment, representative of independent two experiments with three to four mice (**a**–**c**), two experiments with two to three mice (**d**).

To clarify whether skin is the primary cite for the increase of Vγ4^+^ γδ T cells, we took the time course of γδ T cell number in the skin as well as in the skin draining lymph nodes (dLNs) at one and three weeks after HFD feeding. We also examined the phenotype in IMQ-induced psoriasis model at each time point. HFD-fed mice exhibited significantly increased weight gain at 3 weeks, however, there were no significant differences in the ear swelling responses in the psoriasis model and the number of Vγ4^+^ γδT cells in the skin between ND and HFD-fed mice (Fig. 5a,b). On the other hand, HFD-fed mice exhibited significantly increased number of Vγ4^+^ γδT cells in the skin dLNs at 3 weeks (Fig. 5c). In addition, the number of Vγ4^+^ γδT cells was significantly increased in the peripheral blood at 10 weeks after HFD-feeding (Fig. 5d). These results suggest that the increase of Vγ4^+^ γδT cells in the skin observed at 10 weeks after HFD-feeding may be the secondary effects through the HFD-induced systemic increase of Vγ4^+^ γδT cells.

### HFD induces γδ T cell-recruiting chemokines in the skin

**Figure 6:**
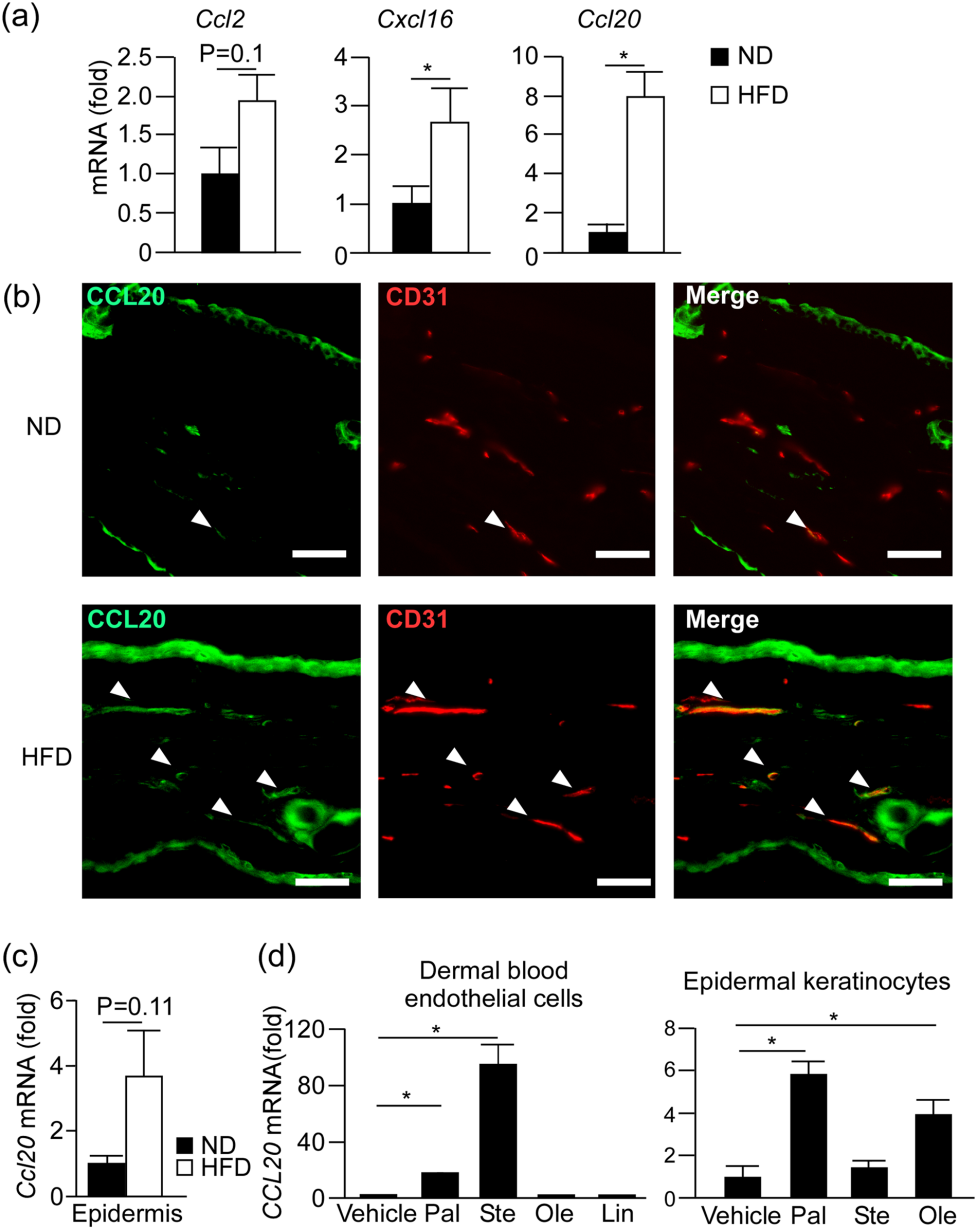
Fatty acid induces *Ccl20* expression from dermal blood endothelial cells (BECs) and epidermal keratinocytes. (**a**) Fold induction of *Ccl2*, *Cxcl16*, *Ccl20* mRNA in the dermis of ND- or HFD-fed mice in the steady state, as analyzed by qRT-PCR. Results are presented relative to those of ND. The average mRNA expression levels in ND-fed mice are set as 1. (**b**) Immunohistochemical staining for CCL20 (green) and CD31 (red) in the ear skin of steady state ND and HFD-fed mice. Arrowheads in the panels indicate CCL20^+^CD31^+^ cells. Scale bars = 50 μm. (**c**) Fold induction of *Ccl20* mRNA in the steady state epidermis, as analyzed by qRT-PCR. Results are presented relative to those of ND. The average mRNA expression level in ND-fed mice is set as 1. (**d**) Fold induction of *CCL20* mRNA expression in human dermal BECs and epidermal keratinocytes cultured with or without fatty acids for 24 h. Results are presented relative to those of the vehicle-treated group. The average mRNA expression levels in ND-fed mice are set as 1. Pal: palmitic acid, Ste: stearic acid, Ole: oleic acid, Lin: linoleic acid. Results are expressed as the mean ± SEM. *p*-values were obtained by (**a**,**c**) Mann-Whitney-U-test and (**d**) one way ANOVA. *p ≤ 0.05. Data are pooled from two experiments with four mice (**a**), two experiments with three mice (**c**). Data are from one experiment, representative of three independent experiments with three to four mice (**b**), three experiments performed in triplicate (**d**).

To further examine the mechanisms of γδ T cell accumulation in the skin, we checked the mRNA expression of several chemokines, such as *Ccl2*, *Cxcl16* and *Ccl20*, that facilitate the infiltration of γδ T cells into tissues^22,23^. We found that the mRNA expression of *Cxcl16* and *Ccl20* was significantly increased in the dermis of HFD-fed mice compared with that of ND-fed mice (Fig. 6a). These results suggest that HFD facilitates the accumulation of γδ T cells by inducing γδ T cell-attracting chemokines in dermis.

### Fatty acids induce CCL20 expression from dermal blood endothelial cells (BECs) and keratinocytes

Since it has been reported that CCL20 is one of the most important chemokine for γδ T cell infiltration in the skin^24^, we focused on CCL20 and examined the HFD-induced regulation of CCL20 expression in more detail. We performed an immunohistochemical analysis of CCL20 using mouse ear skin. Consistent with the previous studies^25^, the epidermis and dermal BECs expressed CCL20 **(**Fig. 6b and Supplementary Figure 5 **)**. The expression level in dermal BECs and epidermis showed a tendency to be stronger in HFD-fed mice than in ND-fed mice **(**Fig. 6b, Supplementary Figure 5 **)**. Consistently, the mRNA expression of *Ccl20* in epidermis showed a tendency to be higher in HFD-fed mice than in ND-fed mice, although the increase was not statistically significant (Fig. 6c).

Then, we next tested the effects of several fatty acids on CCL20 production from cultured-primary human dermal BECs and keratinocytes, since HFD contains a high amount of saturated or unsaturated fatty acids, and it was reported that the serum levels of fatty acid were significantly increased in HFD-fed mice compared with ND-fed mice^26^. In dermal BECs, the mRNA expression of *CCL20* was significantly increased by the stimulation with saturated fatty acid such as palmitic acid and stearic acid (Fig. 6d). In keratinocytes, the expression of *CCL20* was significantly induced by the stimulation with palmitic acid and oleic acid (Fig. 6d). These results suggest that some fatty acids in HFD are responsible for the HFD-induced *CCL20* expression in the skin.

## Discussion

Obesity has been suspected as a trigger or exacerbating factor of psoriasis, since epidemiological studies indicate the association between obesity and psoriasis^2–4^. In addition, recent studies suggest that HFD, which potentially induces obesity, is also responsible for the exacerbation of psoriatic dermatitis^13,27^, which is in line with our current findings. We also revealed novel mechanisms of HFD-induced exacerbation of psoriatic dermatitis, such as induction of systemic increase of IL-17-producing γδ T cells and their recruitment to the skin possibly by inducing γδ T cell-recruiting chemokines in the skin.

We consider that the high expression of IL-17A in the skin of HFD-fed mice in basal condition is the key point in the exacerbated IMQ-induced psoriatic dermatitis in HFD-fed mice, because IL-17A can significantly enhance the functions of various proinflammatory cytokines. For example, it has been reported that expression levels of mRNA of proinflammatory cytokines in keratinocytes stimulated with IL-17A plus TNF-α are 2 to 10 times higher compared with those stimulated with IL-17A alone^28^. It has also been reported that injection of IL-17A alone does not induce psoriatic dermatitis *in vivo*, while injection of IL-17A with IMQ treatment induces “exacerbation” of psoriatic dermatitis compared with that induced by IMQ alone^13^. These results suggest that various inflammatory mediators induced by IMQ may be necessary for the development of psoriatic dermatitis by working with IL-17A cooperatively. Vice versa, IL-17A would be essential for the development of psoriatic dermatitis, because the absence of IL-17A alone prevents IMQ-induced psoriatic dermatitis, consistent with clinical data in psoriasis^29^.

Other than these mechanisms, several mechanisms have been proposed as the HFD-induced exacerbation of psoriasis. It has been reported that a HFD drives Th17 cell differentiation by inducing a lipid metabolic kinase^30^. A HFD also activates IL-17-producing innate lymphoid cells in the lung^31^ or CD11c^+^ cells in the skin^16^ through NLRP3 inflammasome stimulation^13^. Hyper-proliferation of keratinocytes is detected in HFD-fed mice of IMQ-induced psoriasis model^12^. Thus, HFD may contribute to the progression of psoriasis by multiple mechanisms.

We also explored which components of HFD induced the exacerbation of psoriatic dermatitis. Our results as well as other previous studies suggest the potential role of fatty acids in the action of HFD. In this study, we revealed that fatty acids induced the mRNA expression of *CCL20* in keratinocytes and BECs. It has been reported that palmitic acids facilitated IL-17-induced activation of keratinocytes *in vitro* ^12^, and that there is a strong correlation between the amount of palmitic acids or oleic acids in serum and the severity of skin inflammation in HFD-induced exacerbation of psoriatic dermatitis^27^. Thus, both saturated fatty acids and unsaturated fatty acids seem to be involved in the effect of HFD on psoriatic dermatitis.

Furthermore, HFDs with different composition of fatty acids might differently influence skin immune cell composition. For example, using a HFD containing saturated fatty acids in different ratio to ours, Vasseur *et al*. reported an increase of IL-17A-producing cells in the steady state skin and exacerbation of IMQ-induced psoriatic dermatitis in HFD-fed mice, which is similar to our results. But increase of IL-17A-producing αβ T cells, not γδ T cells, was prominent in the skin dLNs of their HFD-fed mice^13^, which is different to our findings. These results suggest that difference of fatty acid-composition in HFD may differently affect the immune cell composition. Although we revealed that HFD induced a systemic increase of IL-17A-producing γδ T cells and chemokine production in the skin, it remains unknown how HFD induces such a systemic increase of γδ T cells. In addition, it is uncertain which chemokines actually contribute to the γδ T cell recruitment into skin. In fact, the effect of blocking antibody for CCL20 on γδ T cell infiltration into skin of HFD-fed mice was only partial and did not exert significant effect **(**Supplementary Figure 6 **)**. Decreased egress from skin, or even prolonged survival *in situ* may also be one of the possible mechanisms of γδ T cell accumulation in the skin. These questions should be answered in the future. Nevertheless, our data suggest that the increase of γδ T cell in the skin is a novel mechanism by which HFD promotes murine psoriatic dermatitis.

Another important question is the involvement of γδ T cells in the development of human psoriasis. Although the pathological role of γδ T cells in psoriatic dermatitis is well established in mouse models, the contribution of γδ T cells in human psoriasis has been under debate. Recently, it was reported that human blood contains γδ T cells which produce IL-17A^32^, and that the number of IL-17A producing γδ T cells are decreased in the blood but increased in the skin lesions of human psoriasis^18,32^. These data raised the possibility that γδ T cells may participate in the development of psoriatic dermatitis even in humans.

In summary, we showed a novel mechanism of HFD-induced exacerbation of psoriatic dermatitis. Our results highlighted the possibility of HFD as a factor that links obesity and psoriasis. Elucidation of the mechanistic link may lead to the development of a new therapeutic modality for psoriasis.

## Materials and Methods

### Mice

C57BL/6 mice and *ob*/*ob* mice were purchased from SLC (Shizuoka, Japan). IL-17A-deficient mice^33^ were kindly provided by Prof. Yoichro Iwakura in Tokyo University of Science. All experiments were performed using male mice. All experimental protocols were approved by the Animal Experimentation Committee of Kyoto University and all animal experimental procedures were performed according to the Animal Protection Guidelines of Kyoto University.

### Diet intervention

Starting at 4 weeks of age, male mice were fed with a ND or HFD for 10 weeks, which provided 60% of energy in the form of fat (D12492; Research Diets, New Brunswick, NJ). The 94% of fatty acids in the HFD were composed of palmitic acid (20%), stearic acid (11%), oleic acid (34%), and linoleic acid (29%).

### IMQ-induced psoriasis model

Mice were treated daily for up to 5 d on each ear with 10 mg of 5% IMQ-containing cream (Beselna Cream; Mochida, Tokyo, Japan). Ear thickness was measured daily using digital calipers (Mitutoyo, Kanagawa, Japan).

### Antibodies and flow cytometry

Anti-mouse CD45 (30-F11) and IL-17A (TC11-18H10) antibodies were obtained from BD Biosciences (Franklin Lakes, NJ). Anti-mouse CD11b (M1/70), CD11c (N418), TCRγδ (eBioGL3), and Ki67 (SolA15) antibodies were purchased from eBioscience (San Diego, CA). Anti-mouse F4/80 (BM8), Ly-6G (1A8), MHC class II (M5/114.15.2), TCRβ (H57-597), and Vγ4 (UC3-10A6) antibodies were purchased from BioLegend (San Diego, CA). For intracellular staining, cells were stimulated for 3 h with 50 ng/ml PMA (phorbol myristate acetate; Sigma-Aldrich, St Louis, MO) and 1 μg/ml ionomycin (Wako, Osaka, Japan) in the presence of 10 μg/ml brefeldin A (Sigma-Aldrich) or put 10 μg/ml brefeldin A in the collagenase solution, and then fixed and permeabilized with Cytofix/Cytoperm buffer (BD Biosciences). Flow cytometry was performed using LSRFortessa (BD Biosciences) and analyzed with FlowJo (TreeStar, San Carlos, CA).

### Single cell preparation from ear skin

The ear splits were minced and digested with 1000 U/ml collagenase type II (Worthington Biochemical, Lakewood, NJ) containing 0.1% DNase I (Sigma-Aldrich) for 60 min at 37 °C. The cell suspensions were filtered with 40 μm of cell strainer.

### Histology and immunohistochemistry

For histological examination, tissues were fixed with 10% formalin in phosphate-buffered saline, and were embedded in paraffin. Sections with a thickness of 5 μm were prepared and were stained with hematoxylin and eosin. Means of epidermal thickness were calculated based on three random site measurements. For Immunohistochemical staining, samples were immersed in 1% paraformaldehyde (Nacalai Tesque, Kyoto, Japan) overnight at 4 °C, embedded in OCT compound (Sakura, Torrance, CA), frozen, and then sectioned. After treatment with Image-iT FX Signal Enhancer (Life Technologies), the sections were incubated with anti-mouse CCL20 (AF760, R&D Systems) overnight at 4 °C and then with Alexa Fluor 488 anti-goat IgG (Life Technologies) and Alexa647 anti-mouse CD31 (MEC13.3, BioLegend) for 30 min. As negative controls, we used samples stained with secondary antibody alone. The slides were mounted using ProLong Antifade with DAPI (Life Technologies). Images were captured on a fluorescent microscope (BZ-900, Keyence, Osaka, Japan).

### Quantitative PCR analysis

Total RNA was isolated with an RNeasy Mini kit (Qiagen, Hilden, Germany). For epidermal examination, mice ear skin incubated in 0.5 M ammonium thiocyanate (Wako) at room temperature for 30 minutes and separated^34^. For dermal examination, the dermal side of ear splits was saved by surgical knife. cDNA was synthesized with a PrimeScript RT reagent kit and random hexamers according to the manufacturer’s protocol (TaKaRa, Shiga, Japan). A LightCycler 480 and LightCycler SYBR Green I Master mix were used according to the manufacturer’s protocol (Roche, Basel, Switzerland) for quantitative PCR. The primer sequences used in this study were as follows: *Gapdh*, 5′-CAAGCTCATTTCCTGGTATGAC-3′ (forward) and 5′-GATAGGGCCTCTCTTGCTCAG-3′ (reverse); *Ccl2*, 5′-TAAAAACCTGGATCGGAACCAAA-3′ (forward) and 5′-GCATTAGCTTCAGATTTACGGGT-3′ (reverse); *Cxcl16*, 5′-TCCTTTTCTTGTTGGCGCTG-3′ (forward), 5′-CAGCGACACTGCCCTGG-3′ (reverse); *Ccl20*, 5′-ACTGTTGCCTCTCGTACATACA-3′ (forward), 5′-ACCCACAATAGCTCTGGAAGG-3′ (reverse); *Ccr6*, 5′-CCTCACATTCTTAGGACTGGAGC-3′ (forward), 5′-GGCAATCAGAGCTCTCGGA-3′ (reverse); *Il1a*, 5′-GCATGGCATTCTTAGGAGGA-3′ (forward), 5′-CCAAATGCATTGAGTGTGCT-3′ (reverse); *Il6*, 5′-TCTATACCACTTCACAAGTCGGA-3′ (forward), 5′-GAATTGCCATTGCACAACTCTTT-3′ (reverse); *Il17a*, 5′-CTCCAGAAGGCCCTCAGACTAC-3′ (forward), 5′-GGGTCTTCATTGCGGTGG-3′ (reverse); *Il23a*, 5′-GACCCACAAGGACTCAAGGA-3′ (forward), 5′-CATGGGGCTATCAGGGAGTA-3′ (reverse); *Tgfb1*, 5′-CCGCAACAACGCCATCTATG-3′ (forward), 5′-CCCGAATGTCTGACGTATTGAAG-3′ (reverse); *Tnfa*, 5′-TGCCTATGTCTCAGCCTCTTC-3′ (forward), 5′-GAGGCCATTTGGGAACTTCT-3′(reverse); human *GAPDH* 5′-TGCCTATGTCTCAGCCTCTTC-3′ (forward), 5′-GAGGCCATTTGGGAACTTCT-3′(reverse); and human *CCL20* 5′-CGAATCAGAAGCAGCAAGCAA-3′ (forward), 5′-CCGTGTGAAGCCCACAATAA-3′ (reverse). Fold expression was calculated by the ΔΔCT method and *Gapdh*(mouse) or *GAPDH* (human) was used as a reference gene. Results are presented relative to mRNA expression of ND or vehicle.

### Effects of free fatty acids on human dermal BECs and epidermal keratinocytes

Human dermal microvascular endothelial cells (Promo Cell, Heidelberg, Germany) were incubated in endothelial cell growth medium (Promo Cell). N/TERT-1 keratinocytes derived from normal human epidermal keratinocytes and immortalized with the telomerase catalytic subunit were used for experiments. Cells were cultured in keratinocyte serum-free medium (Life Technologies) supplemented with 0.2 ng/ml of epidermal growth factor (EGF), 25 mg/ml of bovine pituitary extract, 0.4 mM CaCl2 and penicillin/streptomycin. Both cells then incubated for 24 h in fresh growth medium containing 2% BSA in the absence or presence of 500 μmol/l palmitic acid, stearic acid, oleic acid and linoleic acid (Nacalai Tesque). These doses are well within the physiological range for free fatty acid concentrations reported for rodents and humans^35^. Each fatty acid was dissolved in ethanol and diluted 1:100 in endothelial cell growth medium containing 2% (wt/vol) fatty acid–free BSA (Calbiochem, San Diego, Calif) before being added to the cells. Control cells received vehicle.

### *In vivo* CCL20 blocking

Intradermal injection of 20 μl PBS containing 20 μg monoclonal anti-mouse CCL20/MIP3α antibody (R&D Systems) or purified rat IgG1, κ isotype control antibody (BioLegend) was performed into ears of ND- or HFD-fed mice for three consecutive days^24^. Then, the ear skin was collected on day 4, digested, and the number of Vγ4^+^ γδ T cells was analyzed by flow cytometry.

### Statistical analysis

All data were statistically analyzed by analysis of Mann–Whitney U test and one-way analysis of variance. Significance for all statistical tests was shown in figures for *p ≤ 0.05. Bar graphs are presented as mean ± standard error of the mean (SEM) and standard deviation (SD).

## Electronic supplementary material

Supplementary Information
