# Supplementary material for: High fat diet exacerbates murine psoriatic dermatitis by increasing the number of IL-17-producing γδ T cells
Source: Sci Rep. 2017 Oct 26;7:14076. doi: 10.1038/s41598-017-14292-1 (PMC5658347; doi:10.1038/s41598-017-14292-1)

Supplementary Information

High fat diet exacerbates murine psoriatic dermatitis by increasing the number of IL-17-producing γδ T cells

Satoshi Nakamizo[1,2], Tetsuya Honda[1], Akimasa Adachi[1], Takahiro Nagatake[3], Jun Kunisawa[3], Akihiko Kitoh[1], Atsushi Otsuka[1], Teruki Dainichi[1], Takashi Nomura[1], Florent Ginhoux[2], Koichi Ikuta[4], Gyohei Egawa[1], and Kenji Kabashima*[1,2,5]

[1]Department of Dermatology Kyoto University Graduate School of Medicine, Kyoto 606-8507, Japan

[2]Singapore Immunology Network (SIgN) and Institute of Medical Biology (IMB), Agency for Science, Technology and Research (A*STAR), 8A Biomedical Grove, IMMUNOS Building, Biopolis, 138648 Singapore

[3]Laboratory of Vaccine Materials, National Institutes of Biomedical Innovation, Health and Nutrition, Osaka 567-0085, Japan

[4]Laboratory of Biological Protection, Department of Biological Responses, Institute for Virus Research, Kyoto University, Kyoto 606-8507, Japan

[5]PRESTO, Japan Science and Technology Agency, 4-1-8 Honcho, Kawaguchi, Saitama 332-0012, Japan

Supplementary Figure Legend

Supplementary Figure 1. The gating strategy of flow cytometry analysis

Flow cytometric analysis of the immune cells in the ear skin of ND. Data are from one experiment, representative of three independent experiments with three to four mice.

Supplementary Figure 2. Flow cytometric analysis of IL-17A-producing γδ T cells in the ear skin of HFD- or ND-fed mice

Flow cytometric analysis of IL-17A-producing γδ TCR^{mid} γδ T cells in the whole ear skin of HFD- or ND-fed mice with or without IMQ treatment for five days. (a) Cells gated on $CD45^{+}$, γδ TCR^{mid}. (b) The percentage of IL-17A producing γδ T cells among the γδ TCR^{mid} γδ T cells. (c) The mean fluorescence intensity (MFI) of IL-17A producing γδ T cells. Samples were collected at 24h after the last IMQ treatment, and the single cell suspensions were stimulated with phorbol myristate acetate (PMA) and ionomycin in the presence of brefeldin A for 3 h before intracellular staining ((a) and (b) left panel (PMA+)). In the data of (b) right panel (PMA-) and (c), ear skin was digested with collagenase in the presence of brefeldin A, and the single cell suspensions were subjected to flow cytometric analysis. Results are expressed as the mean ± SEM. *p*-values were obtained by one-way ANOVA. $*p \leq 0.05$. Data are from one experiment, representative of three independent experiments with three to four mice.

Supplementary Figure 3. IL-17A is required for the exacerbation of HFD-induced psoriatic dermatitis.

(a) Comparison of body weight between ND- or HFD- fed Wild type (WT) or IL-17A

deficient (*Il17a*$^{-/-}$) mice. (b, c) Flow cytometric analysis of the T cell subset (b) and the number of dermal γδ T cells (c) in the steady state skin of WT and *Il17a*$^{-/-}$ mice fed with either a ND or HFD. (d) IMQ-induced psoriatic dermatitis in WT or *Il17a*$^{-/-}$ mice fed with either a ND or HFD assessed as change in ear swelling. Results are expressed as the mean ± SEM. *p*-values were obtained by (c) Mann-Whitney-U-test and (d) one-way ANOVA. $*p \leq 0.05$. Data are from one experiment, representative of three independent experiments with three to four mice.

Supplementary Figure 4. The mRNA expression of *Il1a, Il6* and *Tgfb1* in the ear skin

Fold induction of *Il1a, Il6* and *Tgfb1* mRNA in the ear skin of ND- or HFD-fed mice in the steady state, as analyzed by qRT-PCR. Results are presented relative to those of ND. The average mRNA expression levels in ND-fed mice are set as 1. Results are expressed as the mean ± SEM. *p*-values were obtained by Mann-Whitney-U-test. Data are pooled from two experiments with three to four mice.

Supplementary Figure 5. Immunohistochemical analysis of CCL20 expression in the ear skin

Immunohistochemical staining for CCL20 (green), CD31 (red), DAPI (blue) in the ear skin of ND and HFD-fed mice in the steady state. Scale bars = 50 μm. Data are from one experiment, representative of two independent experiments with three mice.

Supplementary Figure 6. Fold change of the number of Vγ4^{+} γδ T cells in the skin after anti-CCL20 neutralizing antibody treatment.

Flow cytometric analysis of Vγ4^{+} $\gamma\delta$ T cells in the whole ear skin of HFD- or ND-fed mice 24 hours after anti-mouse CCL20 antibody treatment for three consecutive days. Results are presented as the fold change relative to the average cell number of isotype antibody-treated group (set as 1), and expressed as the mean ± SEM. *p*-values were obtained by Mann-Whitney-U-test. Data are pooled from two independent experiments with three to four mice.

Supplementary Figure 1

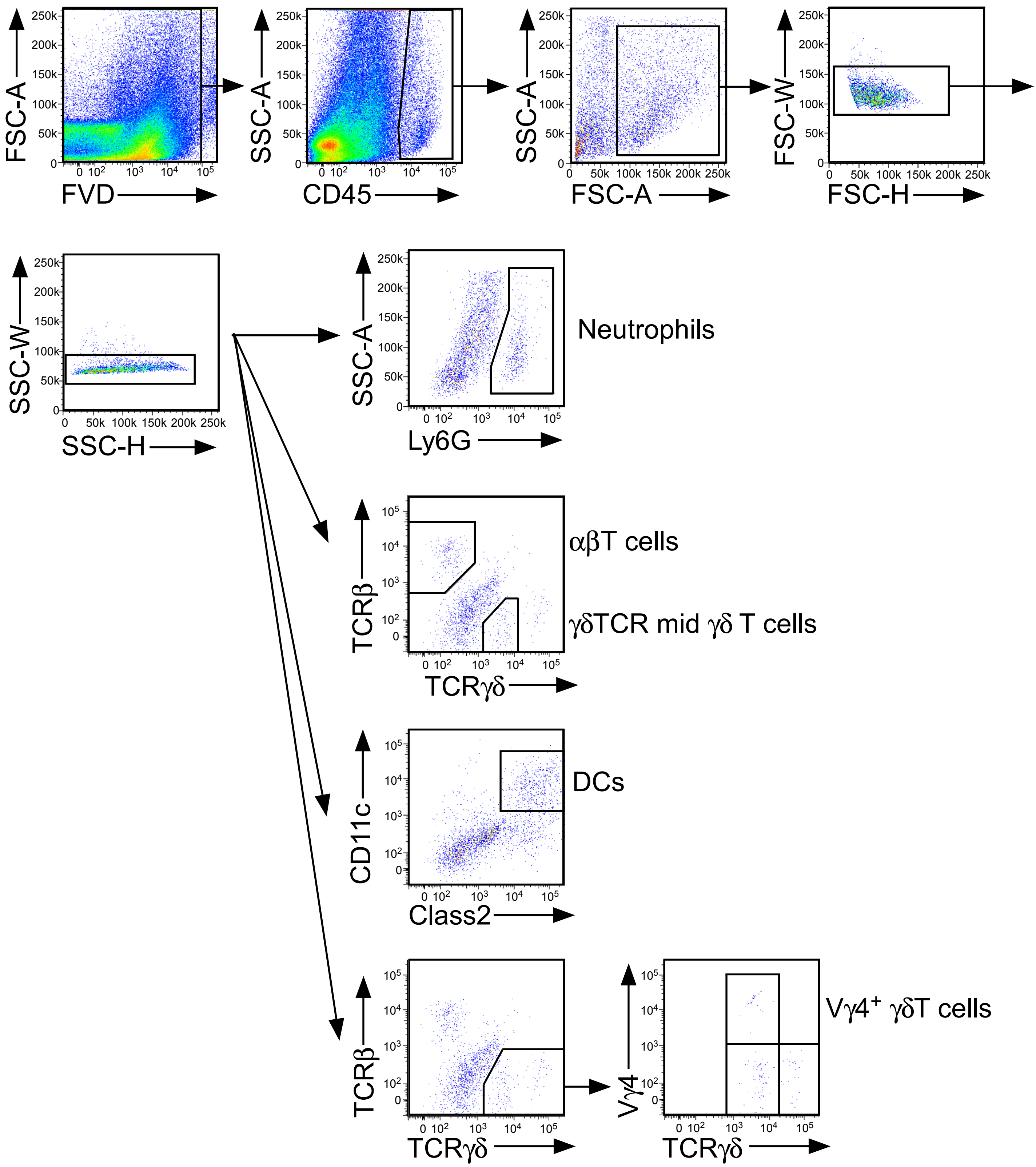

Supplementary Figure 2

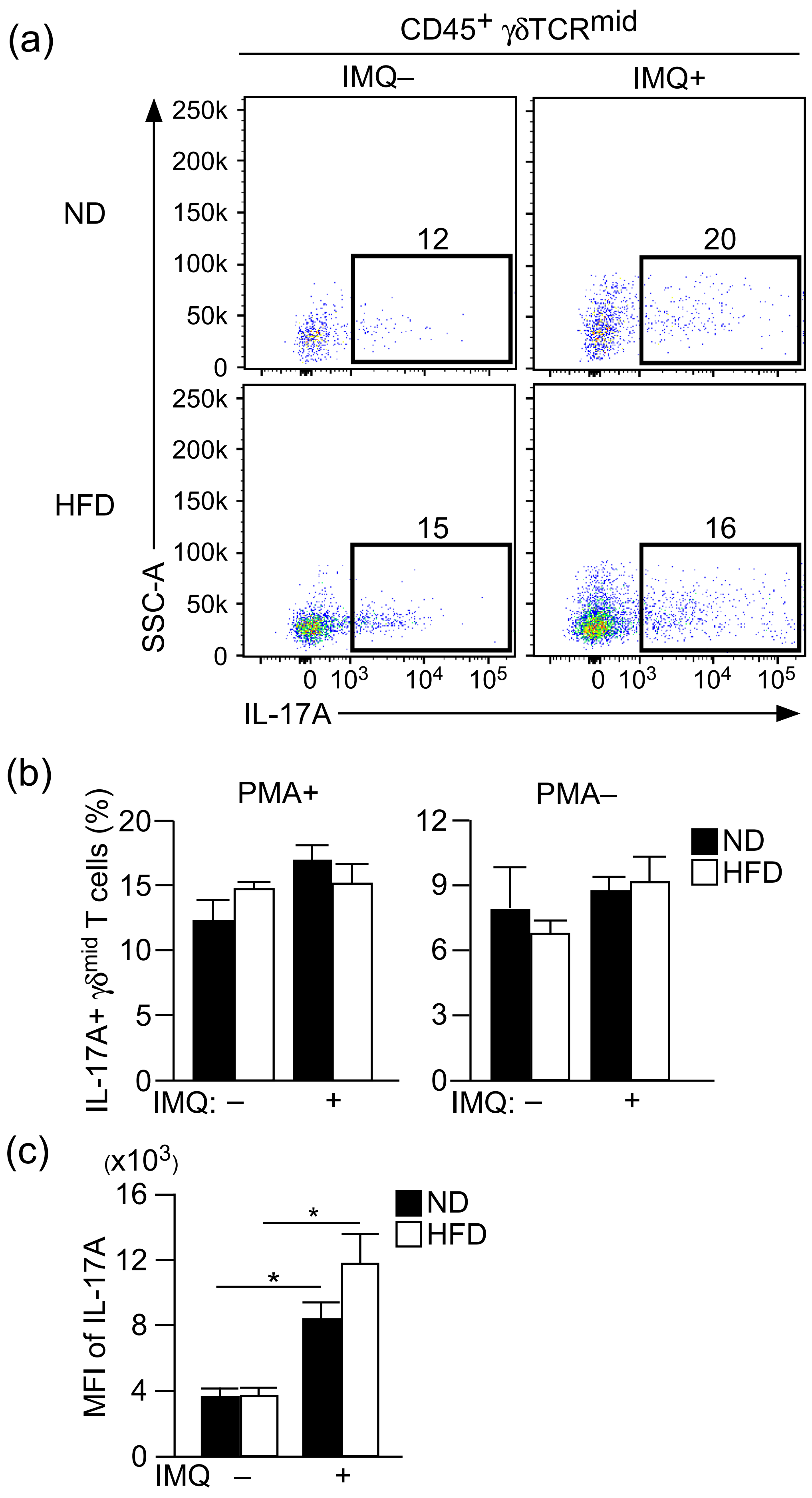

Supplementary Figure 3

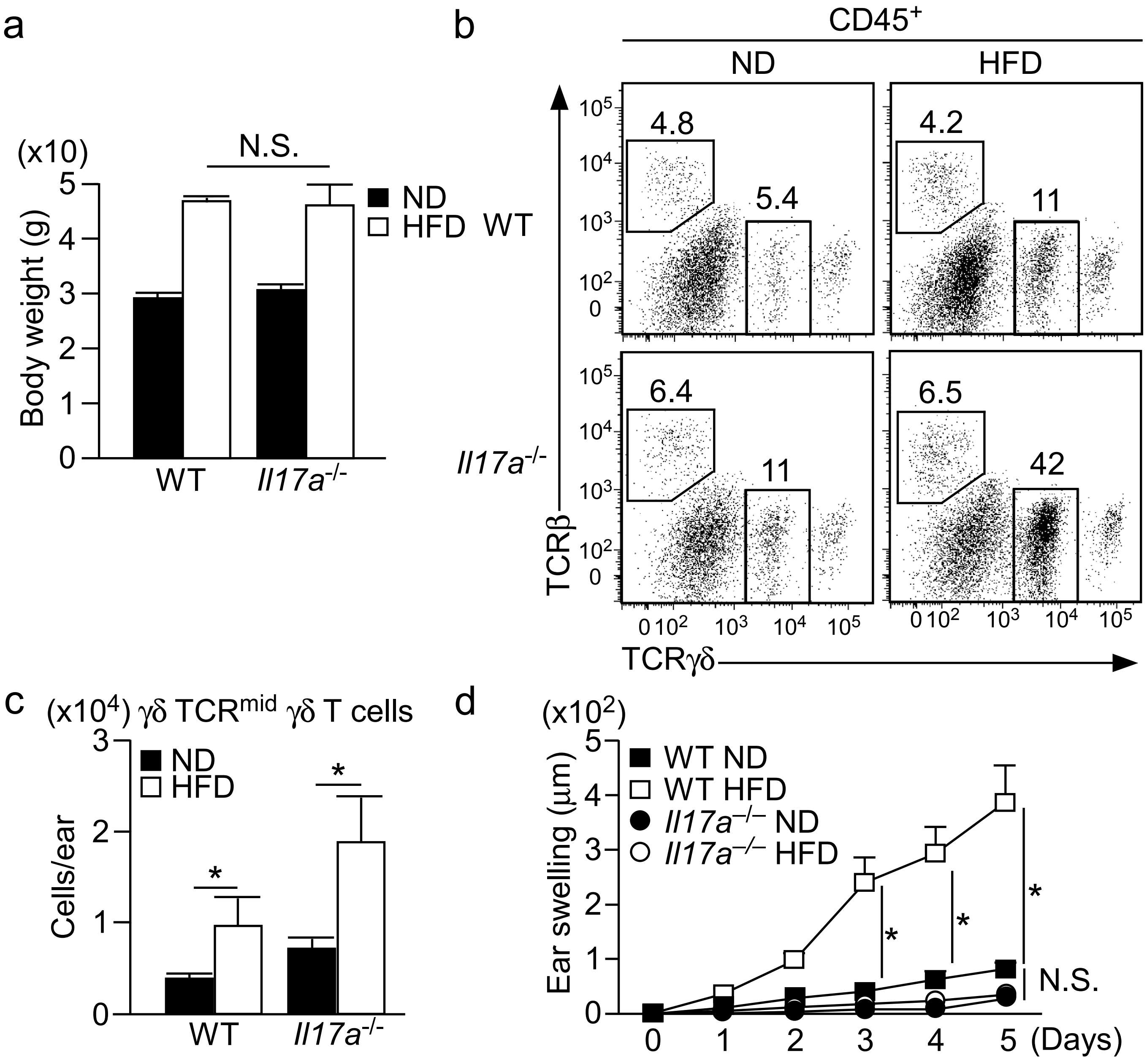

Supplementary Figure 4

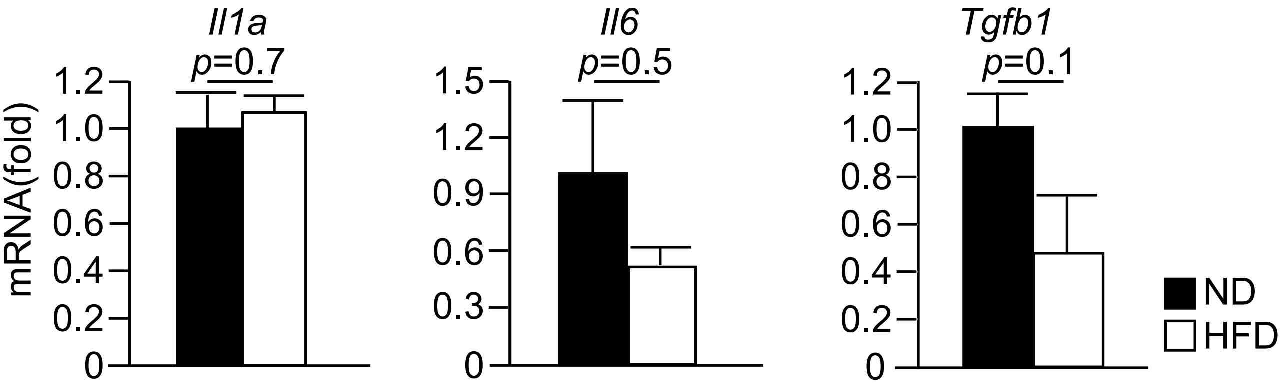

CCR6 was below detection limit

Supplementary Figure 5

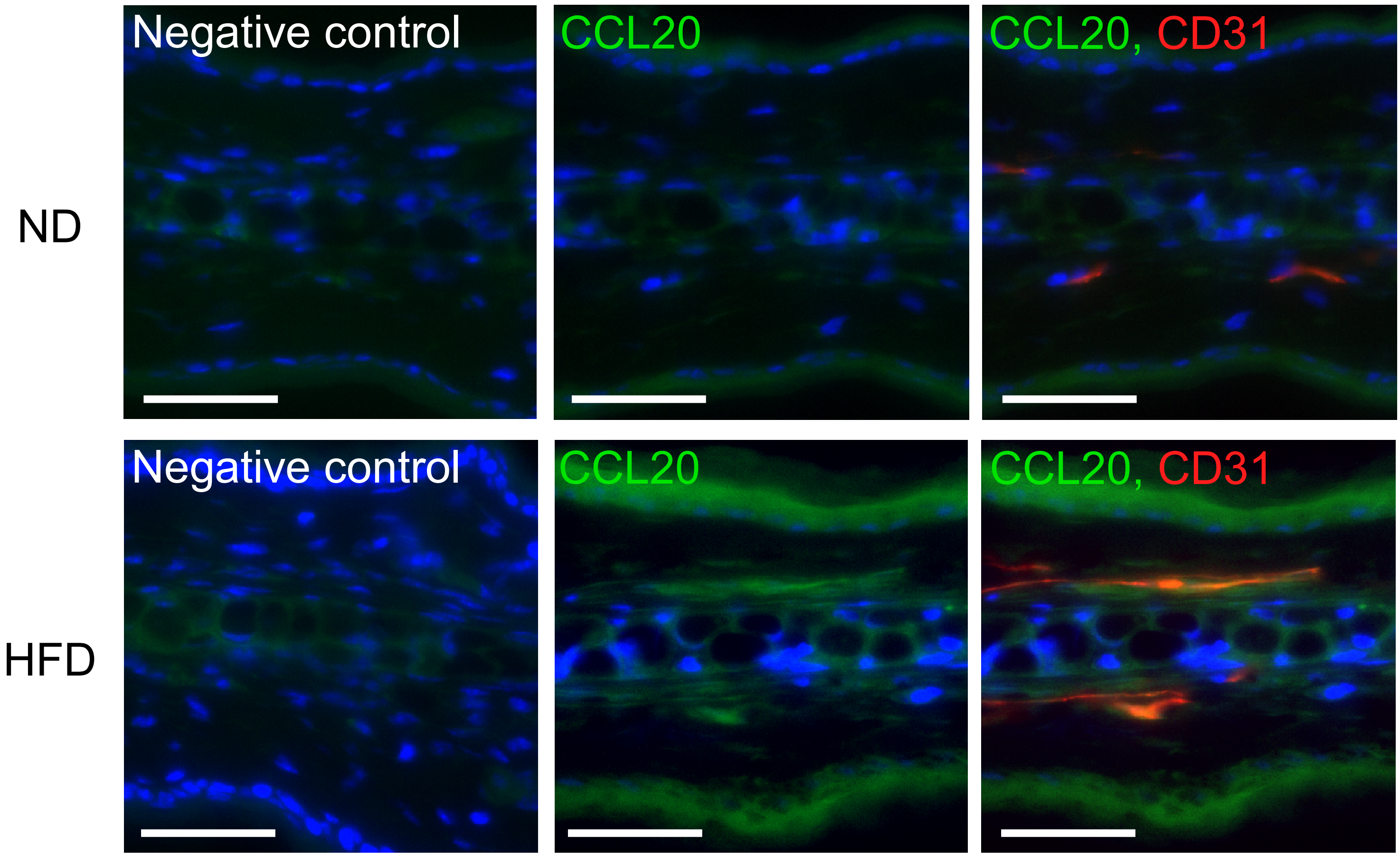

Supplementary Figure 6

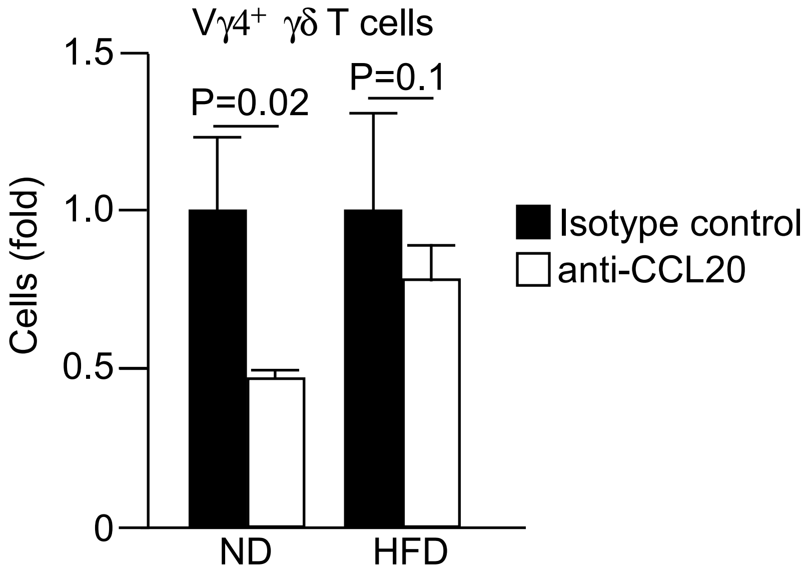

Supplement: Supplementary file 1 — Supplementary Information [file 41598_2017_14292_MOESM1_ESM.pdf]
